# Using a Multiclass Machine Learning Model to Predict the Outcome of Acute Ischemic Stroke Requiring Reperfusion Therapy

**DOI:** 10.3390/diagnostics11010080

**Published:** 2021-01-06

**Authors:** I-Min Chiu, Wun-Huei Zeng, Chi-Yung Cheng, Shih-Hsuan Chen, Chun-Hung Richard Lin

**Affiliations:** 1 Department of Emergency Medicine, Kaohsiung Chang Gung Memorial Hospital, Kaohsiung 83301, Taiwan; outofray@hotmail.com (I.-M.C.); qzsecawsxd@cgmh.org.tw (C.-Y.C.); 2 Department of Computer Science and Engineering, National Sun Yat-sen University, Kaohsiung 804201, Taiwan; ninefour@g-mail.nsysu.edu.tw; 3 Division of Cerebrovascular Diseases, Department of Neurology, Kaohsiung Chang Gung Memorial Hospital, Kaohsiung 83301, Taiwan

**Keywords:** machine learning, acute ischemic stroke, reperfusion therapy, outcome prediction, multiclass classification

## Abstract

Prediction of functional outcome in ischemic stroke patients is useful for clinical decisions. Previous studies mostly elaborate on the prediction of favorable outcomes. Miserable outcomes, which are usually defined as modified Rankin Scale (mRS) 5–6, should be considered as well before further invasive intervention. By using a machine learning algorithm, we aimed to develop a multiclass classification model for outcome prediction in acute ischemic stroke patients requiring reperfusion therapy. This was a retrospective study performed at a stroke medical center in Taiwan. Patients with acute ischemic stroke who visited between January 2016 and December 2019 and who were candidates for reperfusion therapy were included. Clinical outcomes were classified as favorable outcome, intermediate outcome, and miserable outcome. We developed four different multiclass machine learning models (Logistic Regression, Supportive Vector Machine, Random Forest, and Extreme Gradient Boosting) to predict clinical outcomes and compared their performance to the DRAGON score. A sample of 590 patients was included in this study. Of them, 180 (30.5%) had favorable outcomes and 152 (25.8%) had miserable outcomes. All selected machine learning models outperformed the DRAGON score on accuracy of outcome prediction (Logistic Regression: 0.70, Supportive Vector Machine: 0.67, Random Forest: 0.69, and Extreme Gradient Boosting: 0.67, vs. DRAGON: 0.51, *p* < 0.001). Among all selected models, Logistic Regression also had a better performance than the DRAGON score on positive predictive value, sensitivity, and specificity. Compared with the DRAGON score, the multiclass machine learning approach showed better performance on the prediction of the 3-month functional outcome of acute ischemic stroke patients requiring reperfusion therapy.

## 1. Introduction

Ischemic stroke continues to be a devastating disease and a leading cause of disability and mortality worldwide [1,2]. Acute ischemic stroke (AIS), caused by intracranial large vessel occlusion, accounts for the gravest prognosis. Despite reperfusion therapy, which includes intravenous thrombolysis and endovascular thrombectomy, most of the victims spend numerous years living with disability [3,4].

The 3-month functional outcome after an acute stroke event, as determined by the modified Rankin Scale (mRS), has been widely considered a long-term prognosis indicator in stroke patients [5]. Several tools for the prediction of this functional outcome have been developed in the past decades. Machine Learning (ML), an application of artificial intelligence using a computer-based algorithm, is one of these. In recent studies, ML has started to show promising results in predicting the outcomes of stroke patients compared to traditional grading systems [6,7,8,9,10]. Previous studies have focused on the prediction of favorable outcomes, defined as mRS < 3 at 3 months. However, the prediction of severe disability or death, defined as mRS 5–6, should be important as well and separated from mRS 3–4 for the determination of a stroke patient’s treatment course [11,12].

The DRAGON score is a scoring system composed by six different variables evaluated from stroke patients at admission (Table 1). It was created in 2012 to predict both favorable and miserable functional outcomes 3 months after the stroke [12], and it has been validated by several studies with good performance [13,14].

On the other hand, to the best of our knowledge, there is no ML approach for the prediction of the whole spectrum functional outcomes in stroke patients. To assist with the generation of accurate treatment decisions, comprehensive outcome prediction for acute stroke patients is needed. Thus, this study aims to develop a multiclass machine learning prediction model on the 3-month outcome of acute ischemic stroke patients who were candidates for reperfusion therapy at the time of admission.

## 2. Materials and Methods

### 2.1. Study Sample and Data Collection

This was a retrospective study based on records from the Stroke Center of Chang Gung Memorial Hospital, Kaohsiung, Taiwan. The data that support the findings of this study are available from the corresponding author upon reasonable request. This study included AIS patients who were candidates for reperfusion therapy between January 2016 and December 2019. The stroke patients who received reperfusion therapy followed the treatment protocol approved by the Natural Health Insurance of Taiwan and the Guideline of Taiwan Stroke Society. The patients were candidates for endovascular thrombectomy (EVT) if they were above 18 years of age, independent in premorbid daily activities, had a stroke severity as assessed by the NIHSS score of ≥8 and ≤30, and stroke onset ≤8 h in anterior circulation and ≤24 h in posterior circulation infarctions. Patients were considered candidates for intravenous thrombolysis if symptom onset occurred within 4.5 h. Patients who were initially evaluated as eligible for reperfusion therapy but were eventually ruled out from receiving this therapy underwent the conventional treatment plan, including intravenous fluid hydration and antiplatelet therapy in the stroke care unit. These patients were still included in the analysis because we attempted to develop a model that could be applied during initial evaluation. The study protocol was approved by the Institutional Review Board (protocol code 201801687B0A3, date of approval 16 November 2018) and funded by grant CMRPG8I0392 of Chang Gung Medical Foundation.

The clinical variables used in developing the ML model that were collected prior to or at the time of admission included several severity indices, namely the modified Rankin Scale (mRS; range 0–6; high scores indicate a more severe state; a score of 6 indicates death) [15], the National Institute of Health Stroke Scale (NIHSS; range 0–42; high scores indicate more severe neurological deficits) [16], which was evaluated at admission, and the Alberta Stroke Program Early CT Score (range 0–10; low scores indicate a larger ischemic area on the target location) [17]. Demographic and clinical characteristics, including age, sex, pre-stroke mRS score, smoking history, alcohol consumption, previous stroke history, hypertension, diabetes mellitus, dyslipidemia, atrial fibrillation, time from symptom onset to treatment, location of infarction brain area, initial vital signs at admission, laboratory tests, and intervention therapy such as EVT and intravenous tissue Plasminogen Activator (tPA), were also collected for inclusion as ML features. EVT and tPA were counted separately in feature selection.

### 2.2. Stepwise Feature Selection

To prevent overfitting the ML models, all collected features went through feature selection based on the forward stepwise method. The stepwise approach uses a sequence of steps to allow features to enter or leave the model one at a time. In this study, the entry and exit criteria were based on mean accuracy.

### 2.3. Machine Learning Algorithms

We used four different ML algorithms in this study: Logistic Regression (LR), Supportive Vector Machine (SVM), Random Forest (RF), and Extreme Gradient Boosting (XGB). LR is an ML algorithm used for classification problems. It is a predictive analysis algorithm based on the concept of probability that uses the sigmoid function as its cost function. SVM is also an ML model used for classification and regression analysis. Compared to LR, SVM constructs a set of hyperplanes in a higher dimensional space that creates the largest distance to the nearest training data point of any class. The larger the distance of the margin achieved, the lower the generalization error [18]. RF operates by constructing multiple decision trees at training and outputting a classification based on the mean prediction of individual trees [19]. XGB is a scalable end-to-end tree boosting system proposed for sparse data and weighted quantile sketch for approximate tree learning. It was developed to solve real world-scale problems using a minimal amount of resources [20].

### 2.4. Outcome Prediction and Statistical Analysis

The included patients were divided into a training set and a testing set with 10-fold cross-validation. We determined the appropriate parameters of the model with the training set and evaluated their performance with the testing set. The ML models were trained to predict, based on the selected parameters, patients’ functional outcome 3 months after the stroke event and to classify the outcome in one of three categories, which were favorable outcome (defined as mRS 0–2), intermediate outcome (defined as mRS 3–4), and miserable outcome (defined as mRS 5–6). We used the DRAGON score (Table 1) as a comparison for outcome prediction by the selected ML models. The performance measurements included accuracy ((true positives + true negatives)/total sample), positive predictive value (PPV) (true positives/(true positives + false positives)), sensitivity (true positives/(true positives + false negatives)), and specificity (true negatives/(true negatives + false positives)). Statistical analyses were performed using Python 3.8 with Scikit-learn 0.22.2 package [21].

## 3. Results

A total of 617 patients who met the inclusion criteria were assessed for eligibility. After excluding five patients with unavailable mRS at 3 months and 22 patients with missing data, 590 patients were included in the ML analysis. The mean age of those 590 patients was 67.9 ± 12.4 years, and 357 (60.5%) were male. The median onset to treatment time was 158 min, and the median NIHSS score was 10. Forty-two percent of patients received intravenous tPA, 29.7% of patients underwent EVT, and 10.8% of patients received both tPA plus EVT. Of the 590 patients, 180 (30.5%) had favorable outcomes and 152 (25.8%) resulted in miserable outcomes. Other demographic characteristics, including initial vital signs, underlying diseases, and reperfusion therapy received, are listed in Table 2. For patients that received EVT, 78.4% had achieved modified treatment in cerebral ischemia score grades of 2b to 3 and 30.2% of them had an mRS score of 0–2 at 3 months after stroke event.

**Figure 1:**
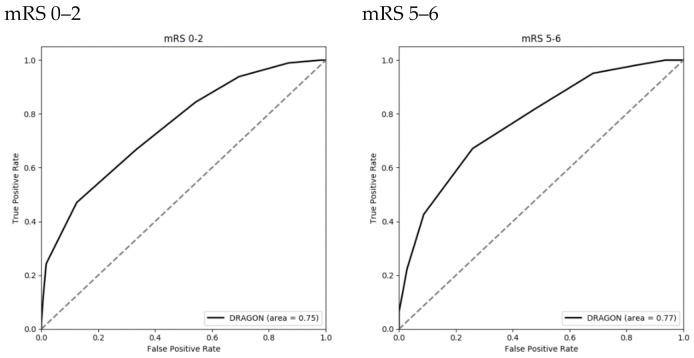
ROC of the DRAGON score to 3-month functional outcome. ROC: receiver operating characteristic curve; mRS: modified Rankin Scale.

Table 3 describes the DRAGON score distribution in the studied sample; the median score was 5 (4–6). Of the total sample, 48 (8.1%) patients had a pre-stroke mRS >1, 273 (46.3%) patients were aged between 65 and 79 years old and 109 (18.5%) were over 80 years old, 69.8% of patients received initial treatment more than 90 min after symptom onset, and 48.1% of patients presented as severe stroke (NIHSS ≥ 16). On correlation with 90-day functional outcome, the DRAGON score showed an Area Under the Curve (AUC) of 0.75 on favorable outcome and 0.77 on miserable outcome (Figure 1).

**Figure 2:**
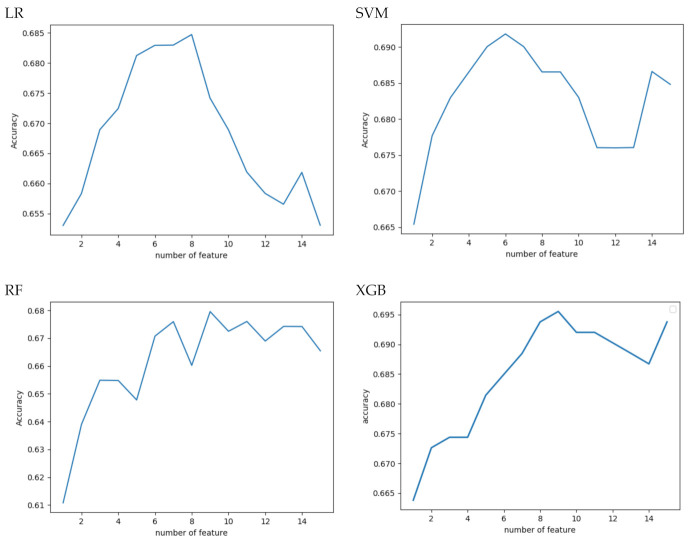
Forward stepwise parameter selection of machine learning models based on average accuracy. LR: Logistic Regression; SVM: Supportive Vector Machine; RF: Random Forest; XGB, Extreme Gradient Boosting.

Regarding ML development, stepwise feature selection based on average accuracy is depicted in Figure 2 for the four different ML algorithms. Based on the results of parameter selection, we used eight, six, nine, and nine parameters for model training in the LR, SVM, RF, and XGB algorithms, respectively. The selected parameters ranked by importance are listed in Table 4. The features selected by all four models were NIHSS at admission, pre-stroke mRS, and EVT, listed according to importance. Other frequent features were diabetes mellitus, age, atrial fibrillation, and onset to treatment time.

**Figure 3:**
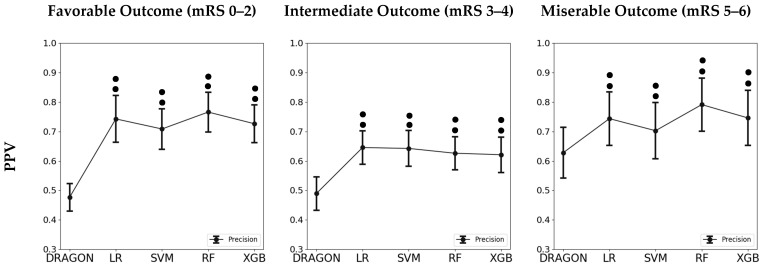

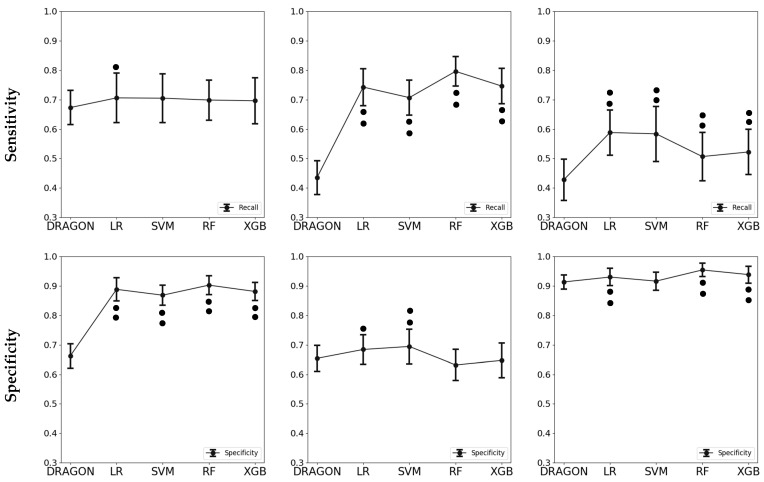
Graphic representation of the results of the one-way ANOVA analysis for different models on predicting each classification of functional outcomes. mRS: modified Rankin Scale; LR: Logistic Regression; SVM: Supportive Vector Machine; RF: Random Forest; XGB: Extreme Gradient Boosting; PPV: Positive Predictive Value. Single dot, *p*-value < 0.05 compared to DRAGON score; Double dot, *p*-value < 0.001 compared to DRAGON score.

In Table 5, we show the results of the one-way ANOVA regarding the comparison of prediction ability between the DRAGON score and the four different ML models. All ML models showed significantly better average accuracy on outcome classification compared to the DRAGON score (*p* < 0.001). All four ML models outperformed the DRAGON score in PPV (*p* < 0.001) and specificity (*p* < 0.001) on predicting favorable outcomes, while statistically significant differences in sensitivity were only found between the DRAGON score and LR (0.67 ± 0.058 vs 0.71 ± 0.084, respectively). Regarding prediction of miserable outcomes, the four ML models showed statistically significant differences with the DRAGON score in PPV (*p* < 0.001) and sensitivity (*p* < 0.001). Except for SVM, the other three ML models also had better specificity. The graphic representation of these findings is depicted in Figure 3.

## 4. Discussion

In this study, we proposed a multiclass ML method for the prediction of the 90-day outcome of patients with AIS who required reperfusion therapy. Improving stroke patients’ outcome is a global concern, especially for those with moderate to severe initial severity. Outcome prediction may be useful in clinical practice to ensure an adequate medical treatment and an individualized rehabilitation program. A wide range of prediction performances of the ML approach have been noted in previous research. Better performance was observed in studies that included all types of acute stroke patients compared to those that considered only large vessel occlusions or patients who received reperfusion therapy. It is conceivable that the more patients with minor stroke are included, the more predictable can the functional outcome be in the near future.

In previous years, the DRAGON score has achieved an acceptable performance in the prediction of both favorable and miserable outcomes from the patients’ initial visit, compared to other indices that mostly focus on forecasting favorable outcomes [13,22]. One study from 2016 even concluded that the DRAGON score predicts stroke outcome more accurately than physicians [14]. In our study, similar results were found, which have been presented in Figure 2. With the majority of patients (75.6%) having presented with moderate to severe symptoms at admission (based on NIHSS), the DRAGON score still had AUC values of 0.75 and 0.77 on predicting favorable and miserable outcomes, respectively.

Nevertheless, the analysis of the commonly used ML models performed in this study showed that ML outperforms the DRAGON score on multiclass classification when there is more admission data available. Except for the sensitivity on favorable outcome prediction, all ML models had better performance on different aspects when assessing classification in three different outcome categories. Similar results have been reported before, as ML has achieved better accuracy than traditional scoring systems on predicting both favorable and not favorable functional outcomes in stroke patients [6,8,9]. This study further proposed a multiclass outcome prediction to show how ML can improve patients’ treatment planning in clinical practice.

The statistical analysis focused on the association between features and outcomes; *p*-values were used as a measure of association. Additionally, we determined the order of importance among features. We used forward stepwise regression not only to control overfitting but also to include the best subsets of features. The basic idea is to impose a constraint on the number of features and then take all the subsets of feature that contain that number, perform ordinary logistic regression, and identify the subset among all combinations that has the best performance (i.e., accuracy in this study). The process results in a list of the best choice one-feature subsets, two-feature subsets, and up to all-feature subsets. The procedure consists of starting with one-feature subsets and then, given the best single feature, finding the second-best feature to add to the evaluation instead of evaluating all possible two-feature subsets. Thus, this model yields the best accuracy for each of the N-feature choices. The idea behind the use of the forward stepwise regression for feature selection, instead of a regression analysis, was to achieve better performance of ML by using feature combinations rather than by mixing statistically significant feature together. By using the stepwise regression feature selection method, we were also able to list the selected features in order, based on their importance to the model (Table 4). Although different ML models disagreed on feature importance in our study, NIHSS, an index of stroke severity based on symptoms, was selected as feature number one in all models. This result was expected because NIHSS has been shown to be independently associated with functional outcome and mortality in stroke patients in numerous studies. EVT was also used in all ML models, although it did not rank within the top five regarding importance. EVT was proven to be beneficial in functional outcome of AIS compared to standard medical care with tPA alone [3]. However, the lack of evidence of its association with 90-day mortality may be the reason for its lower position in the feature selection process. Besides NIHSS and EVT, other features commonly included in the ML models corresponded to the parameters used in the DRAGON score. These features include pre-stroke mRS, diabetes mellitus, age, and onset to treatment time, and all have also been shown to be associated with stroke outcome in several studies over previous decades. We believe that, by knowing which parameters were included in the training, clinicians may have more confidence in using ML models in clinical practice.

This study has several limitations. First, as a retrospective study conducted in a single medical center, the results may have limited generalizability regarding clinical application. Second, with the small sample size, general prediction performance may be over- or under-estimated. However, with the current feature selection method, we will be able to record data more precisely in the future to further improve the prediction ability of the ML method.

## 5. Conclusions

Compared to the DRAGON score, the multiclass ML approach is associated with better accuracy on the prediction of 3-month functional outcomes of AIS patients requiring reperfusion therapy. Among the selected ML models, LR outperformed the DRAGON score on positive predictive value, sensitivity, and specificity in classifying outcomes in three different categories.

## Figures and Tables

**Table 1:** DRAGON score.

Parameter	Category	Points
(Hyper) **D**ense cerebral artery sign or early infarct signs on admission CT head scan	None	0
	Either of them	1
m**R**S > 1, pre-stroke	Both	2
	No	0
	Yes	1
**A**ge	<65 years	0
	65 to 79 years	1
	≥80 years	2
**G**lucose level on admission	<144 mg/dL	0
	>144 mg/dL	1
**O**nset-to-treatment time	≤90 min	0
	>90 min	1
**N**IHSS on admission	0–4	0
	5–9	1
	10–15	2
	>15	3

NIHSS: National Institutes of Health Stroke Scale; mRS: modified Rankin Scale.

**Table 2:** Demographic characteristics of included patients.

Variables	All Patients (*n* = 590)
Mean Age ± SD (years)	67.9 ± 12.4
Male, *n* (%)	357 (60.5)
Onset to treatment in minutes, median (IQR)	158 (74–249)
Clinical Characteristics	
NIHSS, median (IQR)	15 (10–21)
ASPECT, median (IQR)	9 (8–10)
Sugar (mg/dL), mean ± SD	141 ± 57
SBP (mmHg), mean ± SD	161 ± 32
DBP (mmHg), mean ± SD	92 ± 19
Underlying Medical Condition, *N* (%)	
Pre-Stroke mRS > 2	34 (5.8%)
Old stroke	162 (27.5)
Atrial fibrillation	227 (38.5)
Diabetes mellitus	209 (35.4)
Hypertension	463 (78.4)
Dyslipidemia	436 (73.9)
Coronary artery disease	134 (22.7)
Heart failure	81 (13.7)
Smoking	162 (27.5)
Medical Treatment, *N* (%)	
tPA	248 (42.0)
EVT	175 (29.7)
Outcomes, *N* (%)	
mRS 0–2	180 (30.5)
mRS 3–4	258 (43.7)
mRS 5–6	152 (25.8)

NIHSS: National Institutes of Health Stroke Scale; ASPECTS: Alberta Stroke Program Early CT Score; SBP: Systolic Blood Pressure; DBP: Diastolic Blood Pressure; tPA: tissue Plasminogen Activator; EVT: Endovascular Thrombectomy; mRS, modified Rankin Scale; IQR, interquartile rage.

**Table 3:** DRAGON Score of included patients.

Score Variables	Number (%)
DRAGON score, median (IQR)	5 (4–6)
Hyperdense cerebral artery or early infarct sign	229 (38.8%)
Pre-stroke mRS >1	48 (8.1%)
Age (year-old)	
65–79	273 (46.3%)
≥80	109 (18.5%)
Glucose (mg/dL) > 144	176 (29.8%)
Onset to Treatment > 90 min	412 (69.8%)
NIHSS	
0–4	32 (5.4%)
5–9	112 (19.0%)
10–15	162 (27.5%)
≥16	284 (48.1%)

mRS: modified Rankin Scale; NIHSS: National Institutes of Health Stroke Scale.

**Table 4:** Rank of parameter importance after stepwise parameter selection.

Rank	LR	SVM	RF	XGB
1st	NIHSS	NIHSS	NIHSS	NIHSS
2nd	Pre-stroke mRS	DM	DM	SBP
3rd	Onset to treatment	Af	Pre-stroke mRS	CAD
4th	DBP	Pre-stroke mRS	Old stroke	Pre-stroke mRS
5th	Age	Old stroke	Sugar	DM
6th	EVT	EVT	SBP	Af
7th	ASPECT		EVT	EVT
8th	tPA		DBP	Smoking
9th			Af	HTN

LR: Logistic Regression; SVM: Supportive Vector Machine; RF: Random Forest; XGB: Extreme Gradient Boosting; NIHSS: National Institutes of Health Stroke Scale; DM: Diabetes Mellitus; Af: Atrial Fibrillation; mRS: modified Rankin Scale; CAD: Coronary Artery Disease; SBP: Systolic Blood Pressure; DBP: Diastolic Blood Pressure; tPA: tissue Plasminogen Activator; EVT: endovascular thrombectomy; HTN: Hypertension.

**Table 5:** ANOVA analysis of accuracy, positive predictive value, sensitivity, and specificity between models.

Models	DRAGON	LR	SVM	RF	XGB	*p*-Value
Accuracy	0.51 ± 0.033	0.70 ± 0.041 **	0.67 ± 0.039 **	0.69 ± 0.039 **	0.67 ± 0.040 **	<0.001
mRS 0–2						
PPV	0.48 ± 0.047	0.74 ± 0.080 **	0.71 ± 0.068 **	0.77 ± 0.068 **	0.73 ± 0.064 **	<0.001
Sensitivity	0.67 ± 0.058	0.71 ± 0.084 *	0.70 ± 0.083	0.70 ± 0.068	0.70 ± 0.079	0.015
specificity	0.66 ± 0.041	0.89 ± 0.040 **	0.87 ± 0.034 **	0.90 ± 0.032 **	0.88 ± 0.031 **	<0.001
mRS 3–4						
PPV	0.49 ± 0.057	0.65 ± 0.056 **	0.64 ± 0.061 **	0.63 ± 0.057 **	0.62 ± 0.060 **	<0.001
Sensitivity	0.44 ± 0.058	0.74 ± 0.063 **	0.71 ± 0.059 **	0.80 ± 0.050 **	0.75 ± 0.060 **	<0.001
specificity	0.65 ± 0.044	0.68 ± 0.050 *	0.70 ± 0.059 **	0.63 ± 0.053	0.65 ± 0.058	<0.001
mRS 5–6						
PPV	0.63 ± 0.086	0.74 ± 0.091 **	0.70 ± 0.095 **	0.79 ± 0.090 **	0.75 ± 0.093 **	<0.001
Sensitivity	0.43 ± 0.070	0.59 ± 0.077 **	0.58 ± 0.093 **	0.51 ± 0.082 **	0.52 ± 0.077 **	<0.001
specificity	0.91 ± 0.024	0.93 ± 0.029 **	0.92 ± 0.031	0.95 ± 0.022 **	0.94 ± 0.029 **	<0.001

mRS: modified Rankin Scale; LR: Logistic Regression; SVM: Supportive Vector Machine; RF: Random Forest; XGB: Extreme Gradient Boosting; PPV: Positive Predictive Value. *, *p*-value < 0.05 compared with DRAGON score; **, *p*-value < 0.001 compared with DRAGON score.

## Data Availability

Restrictions apply to the availability of these data. Data was obtained from Chang Gung Research Database and Stroke Center of Kaohsiung Chang Gung Memorial Hospital and are available from corresponding author with the permission.

## References

[1] Vos T., Abajobir A.A., Abate K.H., Abbafati C., Abbas K.M., Abd-Allah F., Abdulkader R.S., Abdulle A.M., Abebo T.A., Abera S.F. (2017). Global, regional, and national incidence, prevalence, and years lived with disability for 328 diseases and injuries for 195 countries, 1990–2016: A systematic analysis for the Global Burden of Disease Study 2016. Lancet.

[2] Wang H., Naghavi M., Allen C., Barber R.M., Bhutta Z.A., Carter A., Casey D.C., Charlson F.J., Chen A.Z., Coates M.M. (2016). Global, regional, and national life expectancy, all-cause mortality, and cause-specific mortality for 249 causes of death, 1980–2015: A systematic analysis for the Global Burden of Disease Study 2015. Lancet.

[3] Badhiwala J.H., Nassiri F., Alhazzani W., Selim M.H., Farrokhyar F., Spears J., Kulkarni A.V., Singh S., Alqahtani A., Rochwerg B. (2015). Endovascular Thrombectomy for Acute Ischemic Stroke. JAMA.

[4] Wardlaw J.M., Murray V., Berge E., del Zoppo G., Sandercock P., Lindley R.L., Cohen G. (2012). Recombinant tissue plasminogen activator for acute ischaemic stroke: An updated systematic review and meta-analysis. Lancet.

[5] Eriksson M., Norrving B., Terént A., Stegmayr B. (2008). Functional Outcome 3 Months after Stroke Predicts Long-Term Survival. Cerebrovasc. Dis..

[6] Heo J., Yoon J.G., Park H., Kim Y.D., Nam H.S., Heo J.H. (2019). Machine Learning-Based Model for Prediction of Outcomes in Acute Stroke. Stroke.

[7] Van Os H.J.A., Ramos L.A., Hilbert A., van Leeuwen M., van Walderveen M.A.A., Kruyt N.D., Dippel D.W.J., Steyerberg E.W., van der Schaaf I.C., Lingsma H.F. (2018). Predicting Outcome of Endovascular Treatment for Acute Ischemic Stroke: Potential Value of Machine Learning Algorithms. Front. Neurol.

[8] Bacchi S., Zerner T., Oakden-Rayner L., Kleinig T., Patel S., Jannes J. (2020). Deep Learning in the Prediction of Ischaemic Stroke Thrombolysis Functional Outcomes: A Pilot Study. Acad. Radiol..

[9] Monteiro M., Fonseca A.C., Freitas A.T., Pinho E.M.T., Francisco A.P., Ferro J.M., Oliveira A.L. (2018). Using Machine Learning to Improve the Prediction of Functional Outcome in Ischemic Stroke Patients. IEEE/ACM Trans. Comput. Biol. Bioinform..

[10] Lin C.H., Hsu K.C., Johnson K.R., Fann Y.C., Tsai C.H., Sun Y., Lien L.M., Chang W.L., Chen P.L., Lin C.L. (2020). Evaluation of machine learning methods to stroke outcome prediction using a nationwide disease registry. Comput. Methods Progr. Biomed..

[11] Kwon S., Hartzema A.G., Duncan P.W., Min-Lai S. (2004). Disability measures in stroke: Relationship among the Barthel Index, the Functional Independence Measure, and the Modified Rankin Scale. Stroke.

[12] Strbian D., Meretoja A., Ahlhelm F.J., Pitkäniemi J., Lyrer P., Kaste M., Engelter S., Tatlisumak T. (2012). Predicting outcome of IV thrombolysis–treated ischemic stroke patients. Neurology.

[13] Strbian D., Seiffge D.J., Breuer L., Numminen H., Michel P., Meretoja A., Coote S., Bordet R., Obach V., Weder B. (2013). Validation of the DRAGON score in 12 stroke centers in anterior and posterior circulation. Stroke.

[14] Ntaios G., Gioulekas F., Papavasileiou V., Strbian D., Michel P. (2016). ASTRAL, DRAGON and SEDAN scores predict stroke outcome more accurately than physicians. Eur. J. Neurol..

[15] van Swieten J.C.K.P., Visser M.C., Schouten H.J., van Gijn J. (1988). Interobserver agreement for the assessment of handicap in stroke patients. Stroke.

[16] Adams H.P., Davis P.H., Leira E.C., Chang K.C., Bendixen B.H., Clarke W.R., Woolson R.F., Hansen M.D. (1999). Baseline NIH Stroke Scale score strongly predicts outcome after stroke: A report of the Trial of Org 10172 in Acute Stroke Treatment (TOAST). Neurology.

[17] Barber P.A., Demchuk A.M., Zhang J., Buchan A.M. (2000). Validity and reliability of a quantitative computed tomography score in predicting outcome of hyperacute stroke before thrombolytic therapy. Lancet.

[18] Bastanlar Y., Ozuysal M. (2014). Introduction to machine learning. Methods Mol. Biol..

[19] Ho T.K. (1998). The random subspace method for constructing decision forests. IEEE Trans. Pattern Anal. Mach. Intell..

[20] Chen T., Guestrin C. (2016). XGBoost: A Scalable Tree Boosting System. arXiv.

[21] Pedregosa F., Varoquaux G., Gramfort A., Michel V., Thirion B., Grisel O., Blondel M., Prettenhofer P., Weiss R., Dubourg V. (2011). Scikit-learn: Machine Learning in Python. J. Mach. Learn. Res..

[22] Seiffge D.J., Karagiannis A., Strbian D., Gensicke H., Peters N., Bonati L.H., Kotisaari K., Leppa M., Kejda-Scharler J., Tranka C. (2014). Simple variables predict miserable outcome after intravenous thrombolysis. Eur. J. Neurol..

